# Impact of perioperative prognostic nutritional index changes on the survival of patients with stage II/III colorectal cancer

**DOI:** 10.1002/ags3.12826

**Published:** 2024-05-30

**Authors:** Kyota Tatsuta, Mayu Sakata, Tadahiro Kojima, Toshiya Akai, Mikihiro Shimizu, Yoshifumi Morita, Hirotoshi Kikuchi, Yoshihiro Hiramatsu, Kiyotaka Kurachi, Hiroya Takeuchi

**Affiliations:** ^1^ Department of Surgery Hamamatsu University School of Medicine Hamamatsu Shizuoka Japan; ^2^ Center for Clinical Research Hamamatsu University School of Medicine Hamamatsu Shizuoka Japan; ^3^ Division of Surgical Care, Morimachi Hamamatsu University School of Medicine Hamamatsu Shizuoka Japan; ^4^ Department of Perioperative Functioning Care and Support Hamamatsu University School of Medicine Hamamatsu Shizuoka Japan

**Keywords:** colorectal cancer, colorectal cancer surgery, perioperative nutrition changes, postoperative nutrition, prognostic nutritional index

## Abstract

**Aim:**

To assess the impact of perioperative prognostic nutritional index (PNI) changes on prognosis and recurrence after colorectal cancer surgery.

**Methods:**

A total of 475 patients who underwent curative resection for primary colorectal adenocarcinoma and were diagnosed with pathological stage (pStage) II/III were retrospectively reviewed. The patients were divided into two groups: the high group (preoperative PNI ≤ postoperative PNI, *n* = 290) and the low group (preoperative PNI > postoperative PNI, *n* = 185).

**Results:**

The low group exhibited significantly higher recurrence and mortality rates (all *p* < 0.001). Kaplan–Meier analysis showed worse overall and recurrence‐free survival in the low group (all *p* < 0.001). Perioperative PNI changes predicted prognosis and recurrence independent of preoperative nutritional conditions. Subgroup analyses showed better overall survival and recurrence‐free survival in the high group across various parameters, such as patient background, surgical outcomes, adjuvant chemotherapy, and pathological characteristics. Multivariate analysis revealed that the low group based on perioperative PNI changes (hazard ratio [HR]: 5.809, 95% confidence interval [CI]: 3.451–9.779, *p* < 0.001), pathological T stage (HR: 1.962, 95% CI: 1.184–3.253, *p* = 0.009), and pathological N stage (HR: 3.434, 95% CI: 1.964–6.004, *p* < 0.001) were identified as independent predictors of worse overall survival.

**Conclusions:**

Patients with pStage II/III colorectal cancer who demonstrate a lower postoperative PNI levels compared to preoperative had poorer overall survival and recurrence‐free survival. Perioperative PNI changes can serve as useful biomarkers for predicting survival and recurrence.

## Graphical abstract

Patients with pStage II/III colorectal cancer who undergo curative resection and demonstrate lower postoperative prognostic nutritional index (PNI) levels compared to preoperative PNI levels have poorer overall survival and recurrence‐free survival. Therefore, perioperative PNI changes can serve as useful biomarkers for predicting survival and recurrence in these patients.

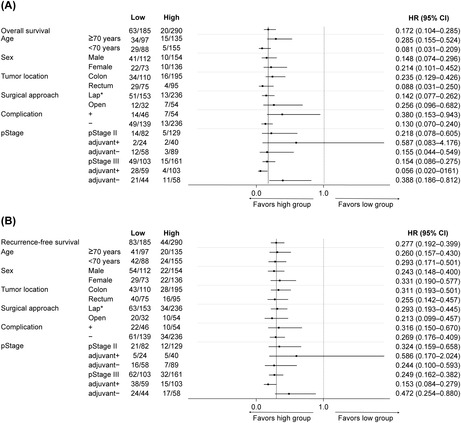

## 1 INTRODUCTION

Colorectal cancer (CRC) is the third most common malignancy worldwide and has the second highest mortality rate among cancer‐related deaths.^1^ In Japan, CRC is the most prevalent cancer and the second‐leading cause of cancer‐related mortality.^2^ Currently, tumor‐node‐metastasis (TNM) classification is the most commonly used method for stratifying CRC prognoses. However, it is well established that there is considerable variation in prognoses among patients classified under the same stage, particularly in stages II and III.^3^, ^4^, ^5^, ^6^ This discrepancy suggests that TNM staging alone does not fully capture the prognostic outcomes of patients. Therefore, there is a need to develop biomarkers that can more accurately stratify patients and identify those with poor prognoses.

Nutritional and inflammatory status markers have been found to be associated with CRC prognosis.^7^ One such marker is the prognostic nutritional index (PNI), which is calculated based on serum albumin levels and total lymphocyte counts. This index can reflect both inflammatory and nutritional status^8^ and has been used in numerous studies.^9^, ^10^, ^11^ Recent retrospective studies focusing on CRC surgery have reported a significant association between low preoperative PNI levels and an increased incidence of postoperative complications, as well as a poor prognosis.^12^, ^13^ However, the definition of low PNI varies among studies, making it difficult to generalize it as a universally applicable biomarker.

To address this issue, our study focused on evaluating the perioperative changes in PNI levels before and after CRC surgery. By examining the magnitude of these changes, there is no need to establish a standard value or definition, potentially allowing for the use of PNI as a biomarker. However, there are little studies investigating perioperative PNI changes before and after CRC surgery. Our hypothesis is that an increase in postoperative PNI levels after CRC surgery compared to preoperative PNI will result in an improved patient prognosis. The purpose of this study was to evaluate the prognostic impact of perioperative PNI changes before and after CRC surgery.

## 2 MATERIALS AND METHODS

### 2.1 Study design and patient population

Between January 2011 and December 2021, 597 patients who underwent curative resection for primary colorectal adenocarcinoma and were diagnosed with pathological stage (pStage) II/III at Hamamatsu University School of Medicine were retrospectively reviewed. The pStage was determined according to the 8th edition of the Union for International Cancer Control TNM classification of malignant tumors.^14^

Patients meeting the following criteria were enrolled in this study: (1) age above 20 years, (2) Eastern Cooperative Oncology Group performance status of 0–1, (3) absence of double cancer, (4) survival for at least 90 days after surgery, and (5) survival and follow‐up for more than 2 years. Patients were excluded from the study if they met any of the following exclusion criteria: interruption of follow‐up within 2 years (*n* = 76), incomplete data (*n* = 30), presence of double cancer (*n* = 9), preoperative chemoradiotherapy (*n* = 4), death within 90 days after surgery (*n* = 3), or emergency surgery (*n* = 1). Finally, a total of 475 patients were included in the study.

### 2.2 Definition of the perioperative PNI levels

**FIGURE 1:**
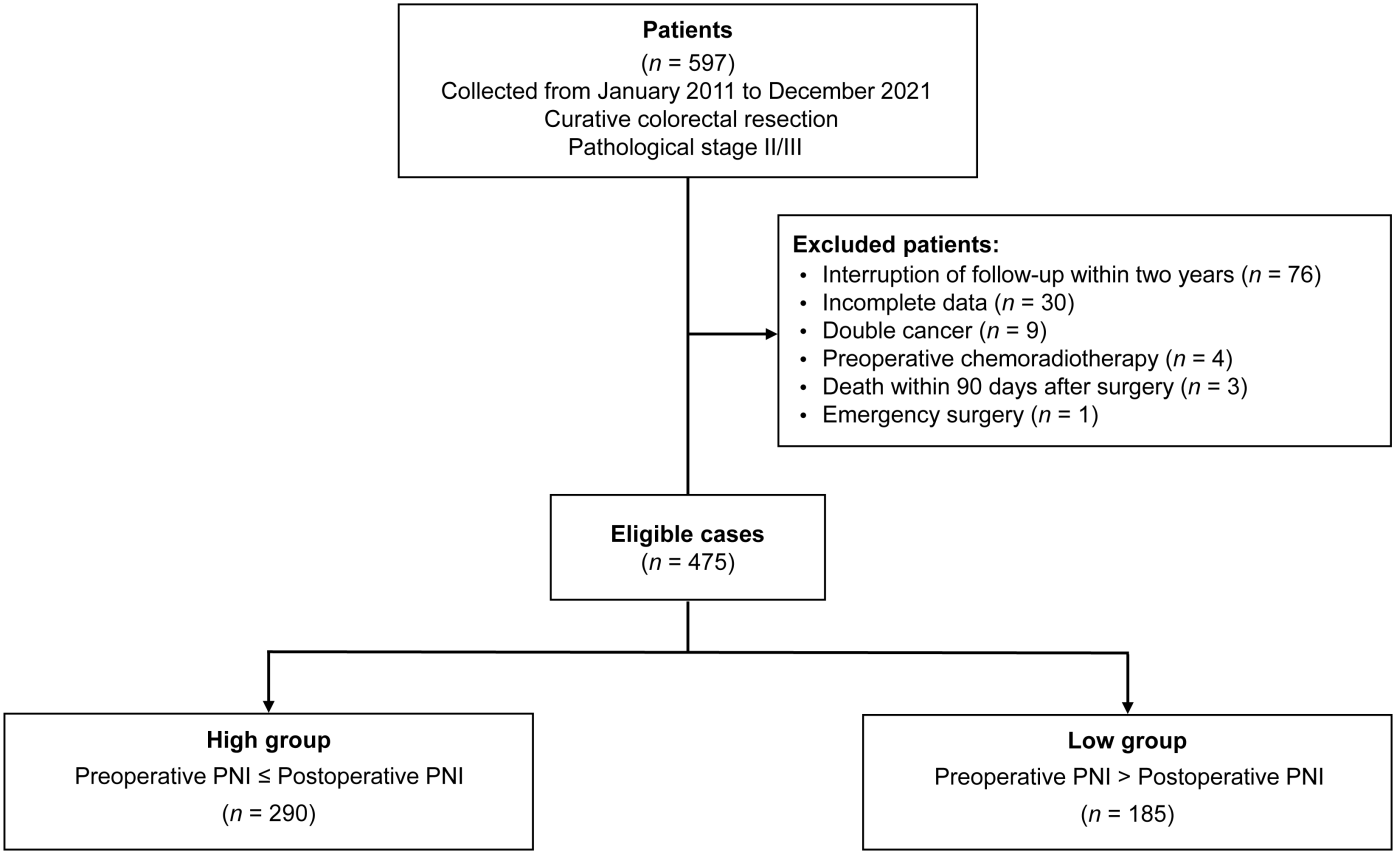
Flow diagram of the present study. PNI, prognostic nutritional index.

The PNI was calculated using the formula: 10 × serum albumin (g/dL) + 0.005 × total lymphocyte counts (per mm^3^).^8^ Preoperative PNI was determined based on blood test results obtained within 2 weeks prior to surgery. If multiple tests were performed, the best result was adopted. Postoperative PNI was determined based on blood test results obtained 1 month after surgery, within a window of 1 week before and after. The same calculation was applied for patients who remained hospitalized for the treatment of postoperative complications during the 1‐month postoperative period. By comparing the preoperative and postoperative PNIs, the patients were divided into two groups: the high group (preoperative PNI ≤ postoperative PNI, *n* = 290) and the low group (preoperative PNI > postoperative PNI, *n* = 185) (Figure 1).

### 2.3 Treatment and postoperative complications

The surgical procedures were performed in accordance with the guidelines set by the Japanese Society for Cancer of the Colon and Rectum.^15^, ^16^ The decision to perform laparoscopic or robot‐assisted surgery was made during team meetings and at the surgeon's discretion. Robotic‐assisted surgery was still in the introductory phase, resulting in a limited number of cases (three cases). Postoperative complications were assessed using the Clavien–Dindo classification, including anastomotic leak, ileus, and others. Complications of Grade 2 or higher were identified as postoperative complications.^17^

### 2.4 Adjuvant therapy and follow‐up

Adjuvant chemotherapy was administered in accordance with the guidelines set by the Japanese Society for Cancer of the Colon and Rectum.^15^, ^16^ Follow‐up visits were scheduled every 3 months during the first 3 years postoperatively, and then every 6 months thereafter. Blood tests and assessments of tumor markers were conducted during each follow‐up visit. Chest–abdomen–pelvic computed tomography was performed every 6 months, and a total colonoscopy was performed annually after surgery. Recurrence‐free survival (RFS) was calculated from the date of surgery to the date of recurrence or death due to any cause. Overall survival (OS) was calculated from the date of surgery to the day of death from any cause, while cancer‐specific survival (CSS) was calculated until death from recurrent cancer. Patients were followed until death or the end of the study period (December 31, 2023). Patients who died from illnesses unrelated to CRC, had interrupted follow‐up, or were still under follow‐up were recognized as censored observations. RFS, OS, and CSS were calculated based on the time until censoring.

### 2.5 Sensitivity analysis

A sensitivity analysis was performed to assess the impact of perioperative PNI changes on prognosis based on preoperative nutritional conditions and systemic inflammatory status. The patients were divided into three groups based on their preoperative PNI levels according to the previous report^8^: <40, 40–45, and >45. Each group was evaluated separately to examine the association between perioperative PNI changes and prognosis. In addition, the reproducibility of the findings was assessed using the Controlling Nutritional Status (CONUT) score, which is an immunonutritional index used to identify undernourished patients in the hospital setting.^18^ In this study, the preoperative nutritional conditions were categorized into three groups based on the CONUT score: 0–1 (normal), 2–4 (mild), and >5 (moderate–severe). Each group was evaluated separately to determine the impact of perioperative PNI changes on the prognosis for different nutritional statuses.

As for preoperative systemic inflammatory status, we used the Glasgow Prognostic Score (GPS)^19^ and C‐reactive protein.^20^ The GPS was divided by scores of 0–2 and CRP by ≥0.5 and <0.5.^21^, ^22^ Data were evaluated in 468 patients (the low group, *n* = 183, and the high group, *n* = 285), excluding data from seven missing cases.

### 2.6 Subgroup analysis

To further assess the impact of perioperative PNI changes on prognosis and recurrence, a subgroup analysis was performed, considering various factors including patient background, surgical outcomes, adjuvant chemotherapy, and pathological characteristics. Pathological characteristics were categorized based on TNM stage and molecular biomarkers for the analysis. Specifically, the focus was on deficient mismatch repair (*dMMR*), which is known to be a favorable prognostic factor in CRC.^23^ At our institution, universal screening for Lynch syndrome by mismatch repair protein immunohistochemistry was started in September 2016. The analysis was limited to dMMR cases identified through this universal screening process. The diagnostic procedures for dMMR were conducted as previously reported.^24^ A total of 28 eligible patients, with 18 in the high group and 10 in the low group, were included in this analysis.

### 2.7 Statistical analyses

The statistical analyses were performed using JMP® 16 (SAS Institute Inc., Cary, NC, USA). Continuous variables were presented as medians and ranges and compared using the Mann–Whitney *U* test. Categorical data were expressed as numbers and frequencies and compared using Fisher's exact test. The RFS, OS, and CSS were calculated using the Kaplan–Meier method and compared using the log‐rank test. Multivariate analyses of survival and recurrence time were performed using Cox proportional hazards regression models. *P*‐values of less than 0.05 were considered statistically significant.

## 3 RESULTS

### 3.1 Clinical characteristics

The characteristics of all study participants are summarized in Table 1. Patient backgrounds were comparable between the two groups. There were no significant differences in surgical outcomes or the rate of patients receiving adjuvant chemotherapy between the two groups.

**TABLE 1:** Patient clinical characteristics.

	Low group (*n* = 185)	High group (*n* = 290)	*p*‐Value
Age, years, median (range)	70 (40–92)	68 (30–95)	0.458
Gender (%)			
Male	112 (60.5)	154 (53.1)	0.129
Female	73 (39.5)	136 (46.9)	
Preoperative BMI, kg/m^2^, median (range)	22.7 (15.7–34.1)	22 (14–38.7)	0.351
ASA‐PS			
3	20 (10.8)	31 (10.7)	1.000
1, 2	165 (89.2)	259 (89.3)	
Tumor markers			
CEA, ng/mL	4.1 (0.8–144)	3.5 (0.5–119.2)	0.637
CA19‐9, U/mL	10 (1–387)	11 (1–2258)	0.460
Tumor location (%)			
Colon	110 (59.5)	195 (67.2)	0.095
Rectum	75 (40.5)	95 (32.8)	
Number of patients who avoided oral intake preoperatively because of colorectal cancer obstruction (%)	5 (2.7)	12 (4.1)	0.460
Clinical T stage			
T1	11 (5.9)	15 (5.2)	0.271
T2	28 (15.1)	35 (12.1)	
T3	100 (54.1)	144 (49.7)	
T4	46 (24.9)	96 (33.1)	
Clinical N stage			
N0	84 (45.4)	118 (40.7)	0.057
N1	66 (35.7)	89 (30.7)	
N2	35 (18.9)	83 (28.6)	
Surgical approach (%)			
Laparoscopy/robotic‐assisted	153 (82.7)	236 (81.4)	0.807
Open	32 (17.3)	54 (18.6)	
LN dissection			
D2	58 (31.4)	86 (29.7)	0.347
D3	127 (68.6)	204 (70.3)	
Complications, C–D grade ≥ 2, (%)			
All complications	46 (24.9)	54 (18.6)	0.108
Anastomotic leakage	12 (6.5)	13 (4.5)	0.401
Ileus	5 (2.7)	11 (3.8)	0.609
Others	29 (15.7)	30 (10.3)	0.089
Pathological stages			
pStage II	82 (44.3)	129 (44.5)	1.000
pStage III	103 (55.7)	161 (55.5)	
Adjuvant chemotherapy			
Yes	83 (44.9)	143 (49.3)	0.348
No	102 (55.1)	147 (50.7)	
Observation period, years, median (range)	4.3 (0.3–10.5)	5.0 (0.9–12.2)	<0.001
Recurrence (%)			
All recurrence	83 (44.9)	44 (15.2)	<0.001
Liver metastasis	29 (15.6)	15 (5.2)	<0.001
Lung metastasis	19 (10.3)	12 (4.1)	<0.001
Peritoneal dissemination	11 (5.9)	6 (2.1)	0.04
Distant lymph node metastasis	11 (5.9)	6 (2.1)	0.04
Others	13 (7)	5 (1.7)	0.005
Death			
All death	63 (34.1)	20 (6.9)	<0.001
Death related to colorectal cancer	54 (29.2)	13 (4.5)	<0.001

Abbreviations: ASA‐PS, American Society of Anesthesiologists physical status; BMI, body mass index; C–D grade, Clavien–Dindo grade; LN, lymph node.

### 3.2 Impact of perioperative PNI changes on recurrence and survival

**FIGURE 2:**
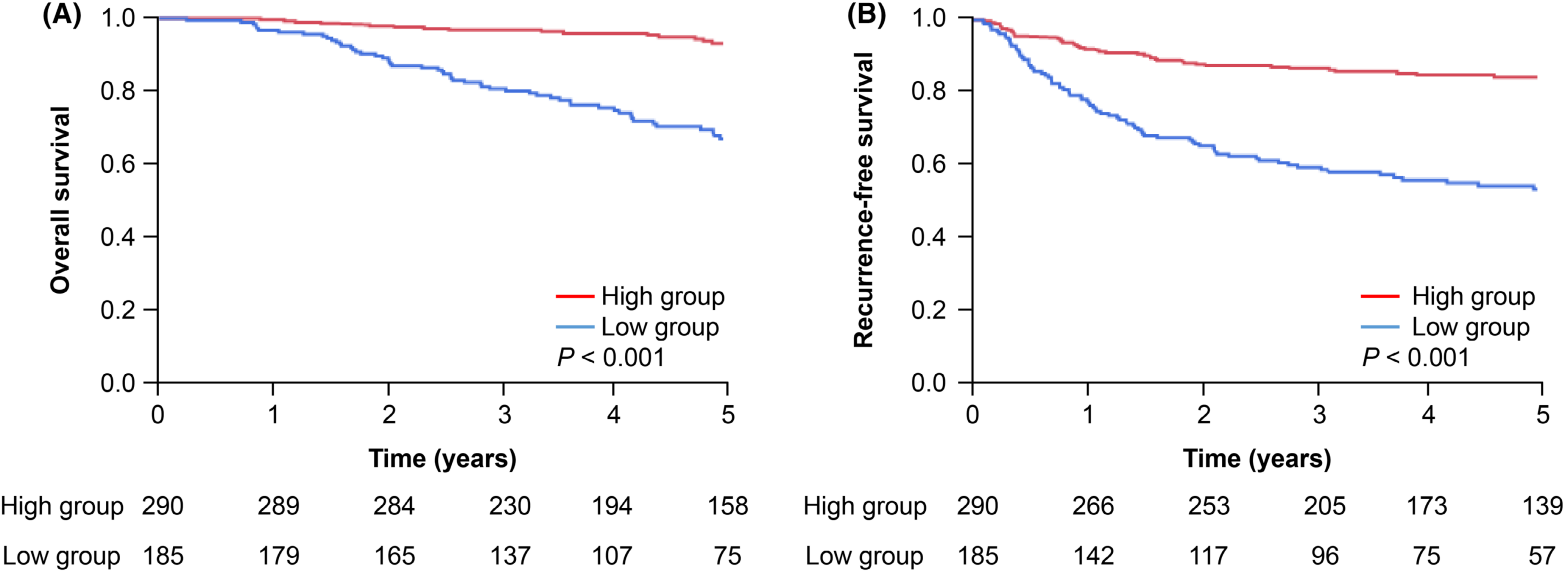
Comparison of overall survival and recurrence‐free survival in Kaplan–Meier analysis. (A) overall survival. (B) recurrence‐free survival.

The recurrence rate was found to be significantly higher in the low group for all types of recurrence, including liver and lung metastases. Moreover, the incidence of death during the observation period was found to be significantly higher in the low group (Table 1). Furthermore, the Kaplan–Meier analysis revealed that both OS and RFS were significantly worse in the low group than in the high group (all *p* < 0.001, Figure 2). Similar results were observed for CSS (Figure S1).

**FIGURE 3:**
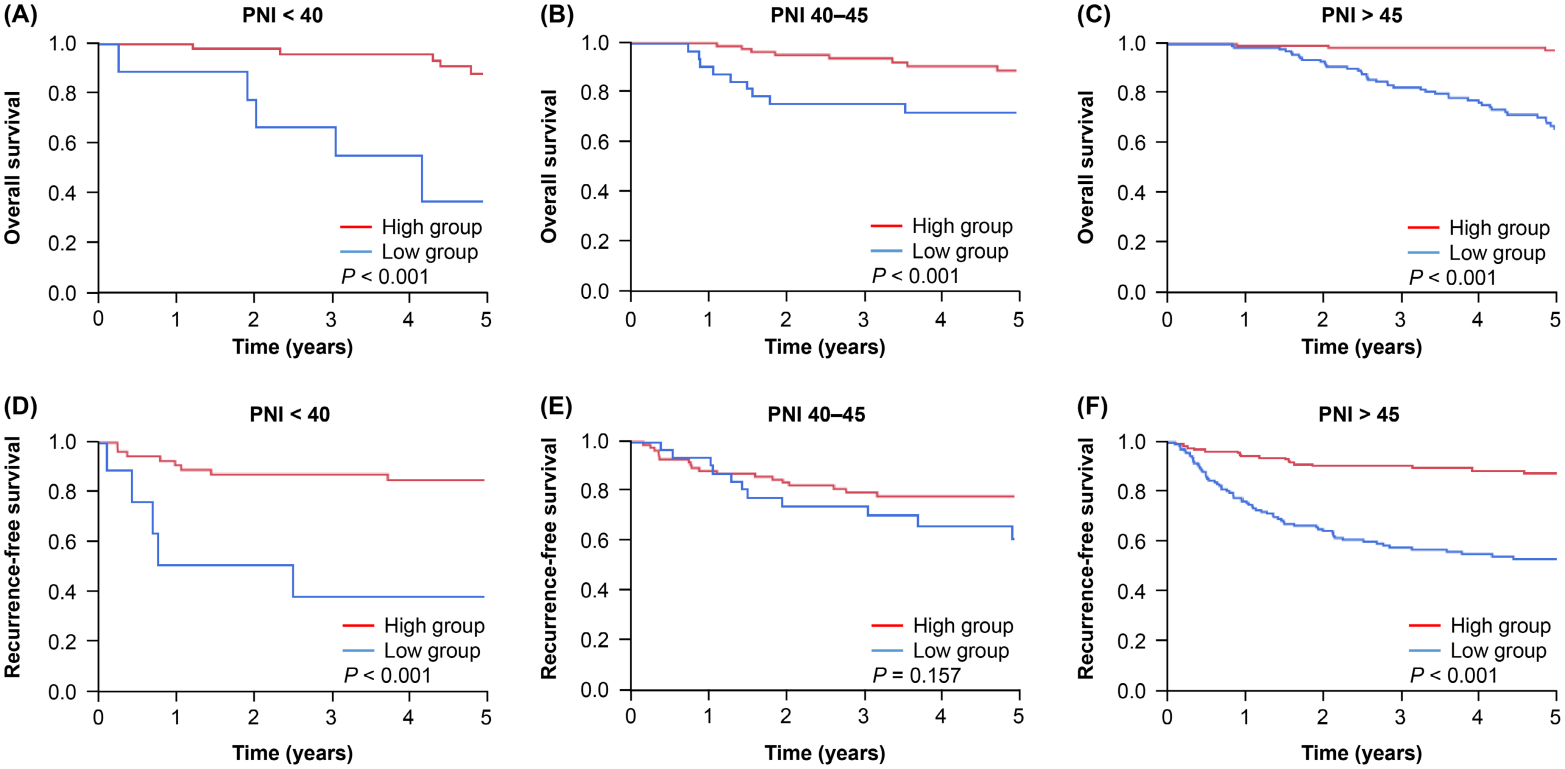
Kaplan–Meier analysis of overall survival and recurrence‐free survival based on the preoperative nutritional conditions. (A–C) Comparison of overall survival categorized based on preoperative PNI levels into <40, 40–45, and >45. (D–F) Comparison of recurrence‐free survival categorized based on preoperative PNI levels into <40, 40–45, and >45. PNI, prognostic nutritional index.

A sensitivity analysis was conducted to further assess the influence of perioperative PNI changes on recurrence and prognosis under different preoperative nutritional conditions and systemic inflammatory status. Patients were categorized into three groups based on their preoperative PNI levels and CONUT scores. These groups were analyzed using the Kaplan–Meier method. Except for the RFS results in patients with PNI levels of 40–45, the high group demonstrated significantly better OS and RFS (Figure 3). Similarly, the analysis using CONUT scores revealed that the high group had significantly better OS and RFS (Figure S2). As for preoperative systemic inflammatory status, the high group was also significantly better for OS, RFS, and CSS than the low group in each GPS and CRP groups (Figures S3 and S4). These findings indicate that regardless of the preoperative nutritional conditions and systemic inflammatory status, perioperative PNI changes can serve as predictors of poor prognosis and high recurrence rates.

### 3.3 Subgroup analysis

**FIGURE 4:**
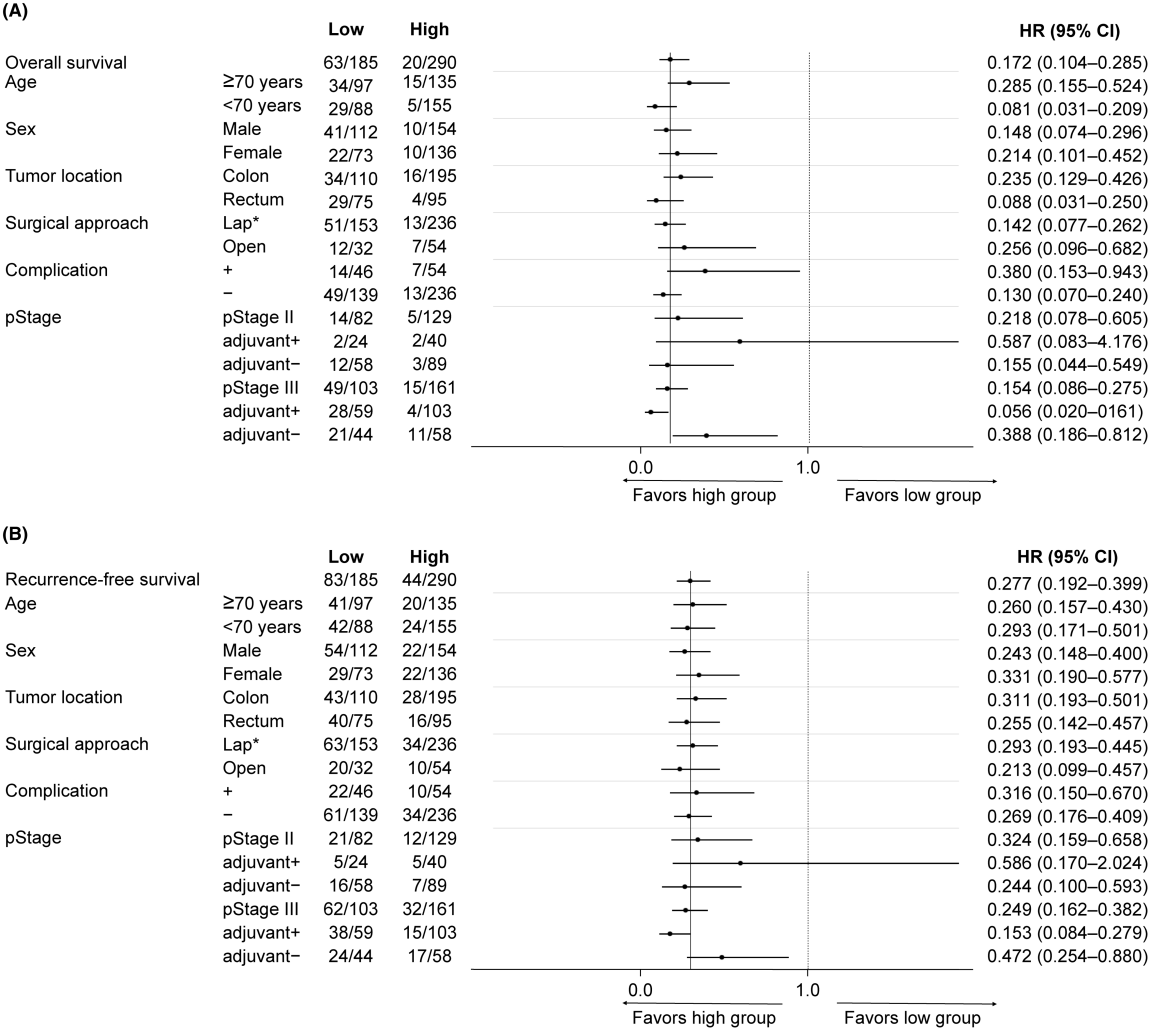
Forest plot of overall survival and recurrence‐free survival based on patient backgrounds, surgical outcomes, adjuvant chemotherapy, and pathological characteristics. (A) Overall survival. (B) Recurrence‐free survival. pStage, pathological stage. *Including three cases of robot‐assisted surgery.

**FIGURE 5:**
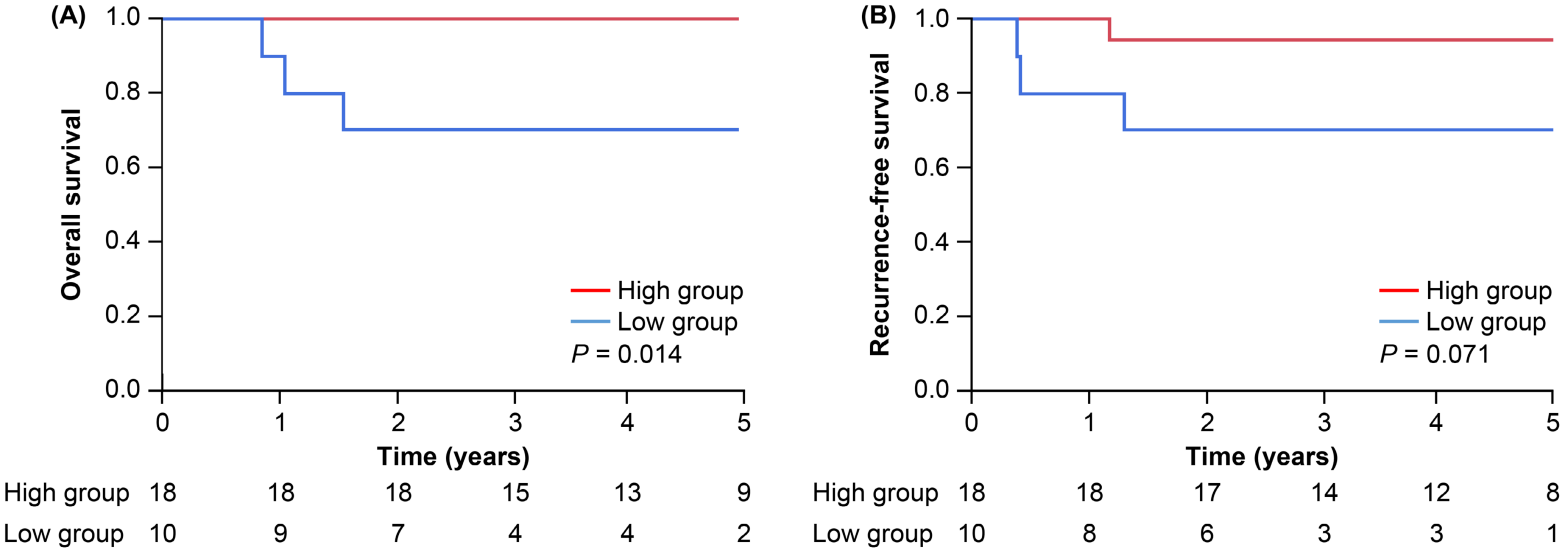
Kaplan–Meier analysis of overall survival and recurrence‐free survival limited to deficient mismatch repair cases. (A) Comparison of overall survival. (B) Comparison of recurrence‐free survival.

To comprehensively assess the impact of perioperative PNI changes on OS and RFS, subgroup analyses were conducted. These analyses focused separately on patient background, surgical outcomes, adjuvant chemotherapy, and pathological characteristics. Across all subgroups except pStage II patients who received adjuvant chemotherapy, patients in the high group exhibited significantly better outcomes in terms of both OS and RFS (Figure 4). Furthermore, when exploring the relationship with molecular biomarkers, the Kaplan–Meier analysis specifically confined to patients with *dMMR*, who are generally predicted to have more favorable outcomes, revealed a significantly worse OS and a trend toward poorer RFS in the low group (Figure 5). Similar results were observed for CSS (Figure S1b,c).

### 3.4 Risk factors for poor prognosis after pStage II/III CRC curative resection

All the variables presented in Table 2 showed potentially significant clinicopathological variables for prognosis in patients with pStage II/III who underwent curative CRC resection. In the multivariate analysis, the low group based on perioperative PNI changes (hazard ratio [HR]: 5.809, 95% confidence interval [CI]: 3.451–9.779, *p* < 0.001), pathological T stage (HR: 1.962, 95% CI: 1.184–3.253, *p* = 0.009), and pathological N stage (HR: 3.434, 95% CI: 1.964–6.004, *p* < 0.001) were identified as independent predictors of worse OS. Similar results were observed for RFS and CSS (Tables S1 and S2).

**TABLE 2:** Results of multivariate analyses showing factors affecting overall survival.

	Multivariable analysis		
	HR	95% CI	*p*‐Value
Age, ≥70 years	1.490	0.912–2.434	0.112
Gender, male	1.264	0.804–1.987	0.310
Low group based on perioperative PNI changes	5.809	3.451–9.779	<0.001
LN dissection, D3	0.798	0.498–1.277	0.346
Histological type, poor/undifferentiated	1.525	0.680–3.419	0.305
Pathological T stage, T4	1.962	1.184–3.253	0.009
Pathological N Stage, N +	3.434	1.964–6.004	<0.001
Adjuvant chemotherapy, yes	0.703	0.429–1.151	0.161

Abbreviations: CI, confidence interval; HR, hazard ratio; LN, lymph node; PNI, prognostic nutritional index.

## 4 DISCUSSION

This retrospective study aimed to evaluate the impact of perioperative PNI changes on the prognosis and recurrence of patients with pStage II/III CRC who underwent curative CRC resection. This study findings revealed that patients categorized in the low group based on perioperative PNI changes demonstrated significantly worse OS and RFS. Importantly, these findings remained consistent regardless of the preoperative nutritional conditions, patient backgrounds, surgical outcomes, adjuvant chemotherapy, and pathological characteristics. To the best of our knowledge, this is the first study to comprehensively evaluate the prognostic impact of perioperative PNI changes before and after CRC surgery. Based on these findings, we propose that perioperative PNI changes serve as a valuable biomarker for predicting prognosis and recurrence of pStage II/III CRC.

The PNI was initially proposed in 1986 as a biomarker for stratifying the safety of resection and anastomosis in gastrointestinal surgery. According to the original definition, a PNI level >45 was considered safe, 40–45 was deemed risky, and <40 was contraindicated.^8^ However, with the aging population and the consequent increase in the age of cancer onset, this definition may not always be applicable.^11^ As a result, recent retrospective studies using the PNI have employed various cutoff values, and even when focusing specifically on CRC resection, different studies have defined a low PNI as below 44.1^25^ or 49.8.^26^ Despite the potential utility of the PNI as a predictor of complications and prognosis, its application as a biomarker in clinical practice presents challenges. The perioperative PNI change that we propose involves only calculating and comparing the PNI before and 1 month after surgery, allowing for straightforward assessment without cutoff values or the need for complex methods. Therefore, we believe that it can serve as a practical and useful biomarker in clinical settings.

Previous studies focusing on perioperative PNI changes are limited. Moreover, similar to studies focusing on preoperative PNI levels, previous studies focusing on perioperative PNI changes also set cutoff values, and each study proposes a different cutoff value. In a study targeting patients who underwent curative resection for Stage II/III CRC, a cutoff value of 43 was used. It has been reported that groups with both preoperative and postoperative low PNI have significantly worse OS.^27^ Another study focusing on patients receiving adjuvant chemotherapy after curative resection for Stage II/III CRC set the cutoff value at 45.6. This study demonstrated that groups with low preoperative and postoperative PNI levels had significantly worse OS, while prognosis improved in groups where the PNI levels were low preoperatively but increased postoperatively.^28^ Similar results have been reported in studies involving patients after esophagectomy.^29^ The consistent finding that patients with lower postoperative PNI levels compared to preoperative PNI levels have a poorer prognosis aligns with our study results. Our proposed perioperative PNI changes could accurately predict recurrence and prognosis without a cutoff value. Therefore, we believe that a cutoff value is no longer necessary when perioperative PNI changes are also used for prognostic and recurrence stratification. In addition, our study shows that, regardless of preoperative nutritional conditions and systemic inflammatory status, patients with lower postoperative PNI levels compared to preoperative PNI levels exhibited significantly worse OS and RFS. These results indicate that there is no bias due to preoperative nutritional conditions and systemic inflammatory status in the calculation of perioperative PNI changes.

Furthermore, defining the timing of perioperative PNI measurement essentially serves as a useful biomarker. In previous studies, the timing of preoperative PNI measurement was defined, but the timing of postoperative PNI measurement varied.^27^, ^28^ Thus, its practical application has been a challenge. Our proposed postoperative PNI measurement period was 1 month after CRC surgery. This minimized the impact of adjuvant chemotherapy. Recent guidelines recommend the initiation of postoperative adjuvant chemotherapy within 2 months after surgery.^14^ If measurements are taken after 1 month postoperatively, they are potentially affected by adjuvant chemotherapy.^29^ We determined this timing of the postoperative PNI measurement to minimize the effect of postoperative adjuvant chemotherapy. This definition has also been used in perioperative PNI studies in patients who underwent esophagectomy. Therefore, it possibly represents a useful biomarker for patients with other cancers.

The results of our subgroup analyses also demonstrate that perioperative PNI changes are applicable to most patient groups undergoing curative resection for pStage II/III CRC. Notably, even in the analysis limited to cases with dMMR, a known favorable prognostic factor in CRC, it was observed that patients with lower postoperative PNI levels exhibited worsened OS and RFS compared with preoperative PNI levels. Molecular biomarkers play a crucial role in the current treatment of CRC.^30^, ^31^, ^32^ Nevertheless, the relationship between PNI and molecular biomarkers has not been explored in previous studies. Due to the limited number of cases in our study, we were unable to examine the prognostically adverse BRAFV600E mutation.^31^, ^33^ Our study contributes significantly to the future research exploring the correlation between molecular biomarkers and nutritional factors. Also, remarkably, perioperative PNI was not associated with OS, RFS, or CSS in pStage II patients who received adjuvant chemotherapy. In contrast, OS, RFS, and CSS were significantly worse in the low group of patients who did not receive adjuvant chemotherapy. These results indicate that in the low group, perioperative PNI may be a biomarker for recommending adjuvant chemotherapy in pStage II patients, in addition to predicting prognosis and recurrence.

Perioperative PNI changes may indicate dramatic alterations in the tumor immune response following curative surgery for colorectal cancer. Inflammatory cytokines derived from cancer cells can activate neutrophil proliferation, suppress lymphocytes, and increase the breakdown of proteins, including albumin.^34^ Therefore, the high group, based on perioperative PNI, could potentially dissociate from the impact of tumor immunity earlier, potentially leading to favorable outcomes. To establish the clinical significance of perioperative PNI changes as a clinically relevant biomarker, validating this hypothesis in future studies is necessary.

The study has some limitations that should be acknowledged. First, it was a retrospective study conducted at a single institution. However, efforts were made to minimize selection bias by including consecutive patients at our institution. Second, the study period spanned a relatively long period of time, during which changes in the treatment of patients, including advancements in surgical techniques, modifications in the adjuvant chemotherapy regimen, and improvements in perioperative management, may occur. Lastly, some biases in the operation and perioperative interventions may exist. Therefore, multi‐institutional prospective studies are required to validate the current study findings.

In conclusion, patients with pStage II/III CRC who undergo curative resection and demonstrate lower postoperative PNI levels compared to preoperative PNI levels have poorer OS and RFS. Therefore, perioperative PNI changes can serve as useful biomarkers for predicting survival and recurrence in these patients.

## AUTHOR CONTRIBUTIONS

Kyota Tatsuta wrote the manuscript, was involved in data collection. Mayu Sakata, Tadahiro Kojima, Kiyotaka Kurachi, and Hiroya Takeuchi designed and conceived the study. Toshiya Akai was involved in data collection. Mikihiro Shimizu was involved in statistical analysis. Yoshifumi Morita, Hirotoshi Kikuchi, Yoshihiro Hiramatsu, and Hiroya Takeuchi critically revised the report and commented on the drafts of the manuscript. All authors have read and approved the final manuscript.

## FUNDING INFORMATION

This study received no funding.

## CONFLICT OF INTEREST STATEMENT

Authors declare no conflict of interests for this article. Hiroya Takeuchi is an editorial board member of *Annals of Gastroenterological Surgery*.

## ETHICAL STATEMENT

Approval of the research protocol: The study design was approved by the Institutional Review Board of Hamamatsu University School of Medicine (IRB number 17‐132) and it conforms to the provisions of the Declaration of Helsinki.

Informed Consent: The requirement for patient consent was waived because of the retrospective nature of the study.

Registry and the Registration No. of the study/trial: N/A.

Animal Studies: N/A.

## Supporting information


Figure S1.

Figure S2.

Figure S3.

Figure S4.

Table S1.

## Data Availability

The datasets used and/or analyzed during the current study are available from the corresponding author on reasonable request.
