# Supplementary figures and images for: Impact of perioperative prognostic nutritional index changes on the survival of patients with stage II/III colorectal cancer
Source: Ann Gastroenterol Surg. 2024 May 30;8(5):817–25. doi: 10.1002/ags3.12826 (PMC11368507; doi:10.1002/ags3.12826)

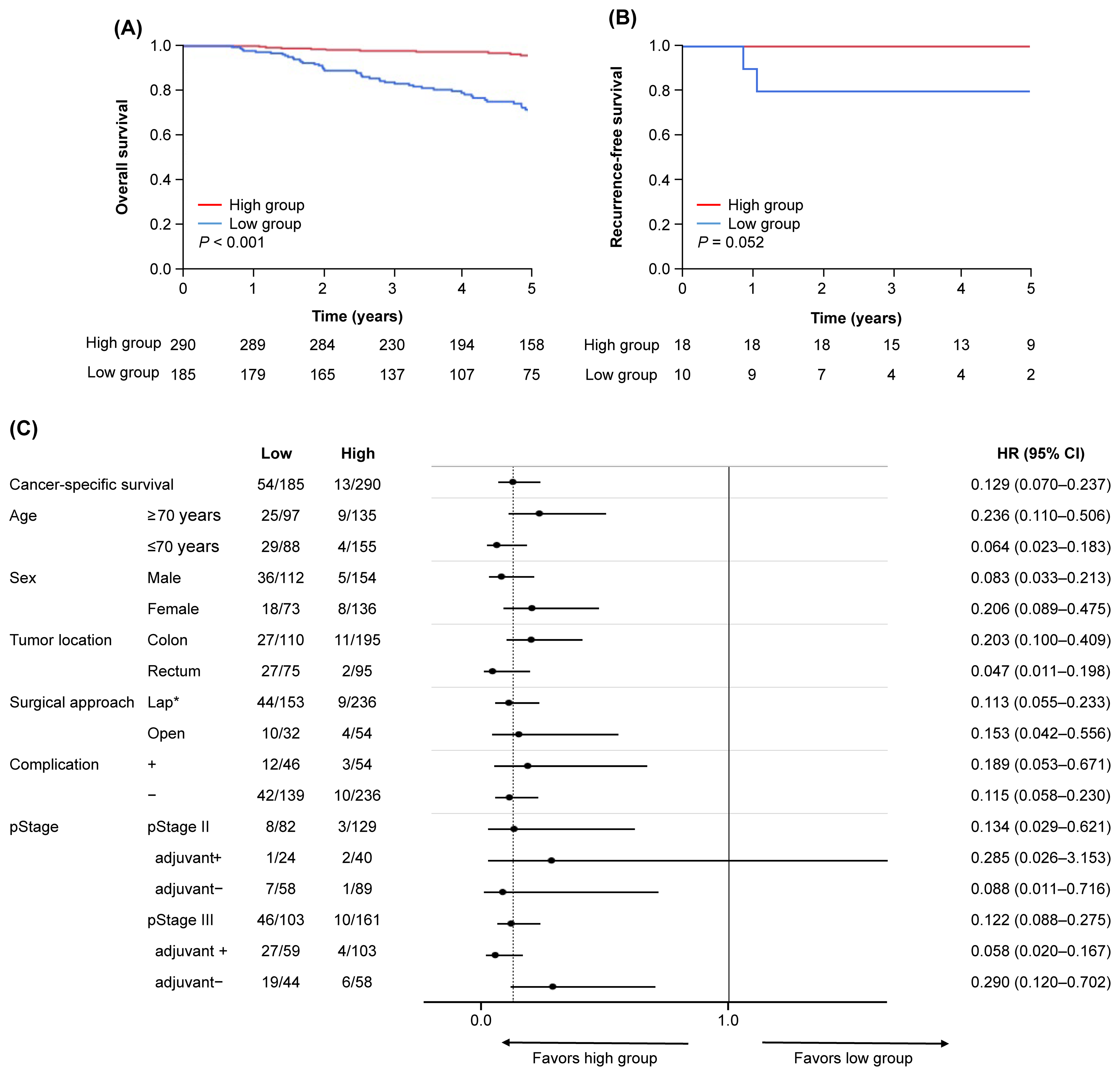

Supplement: Supplementary file 1 — Figure S1. [file AGS3-8-817-s005.tif]

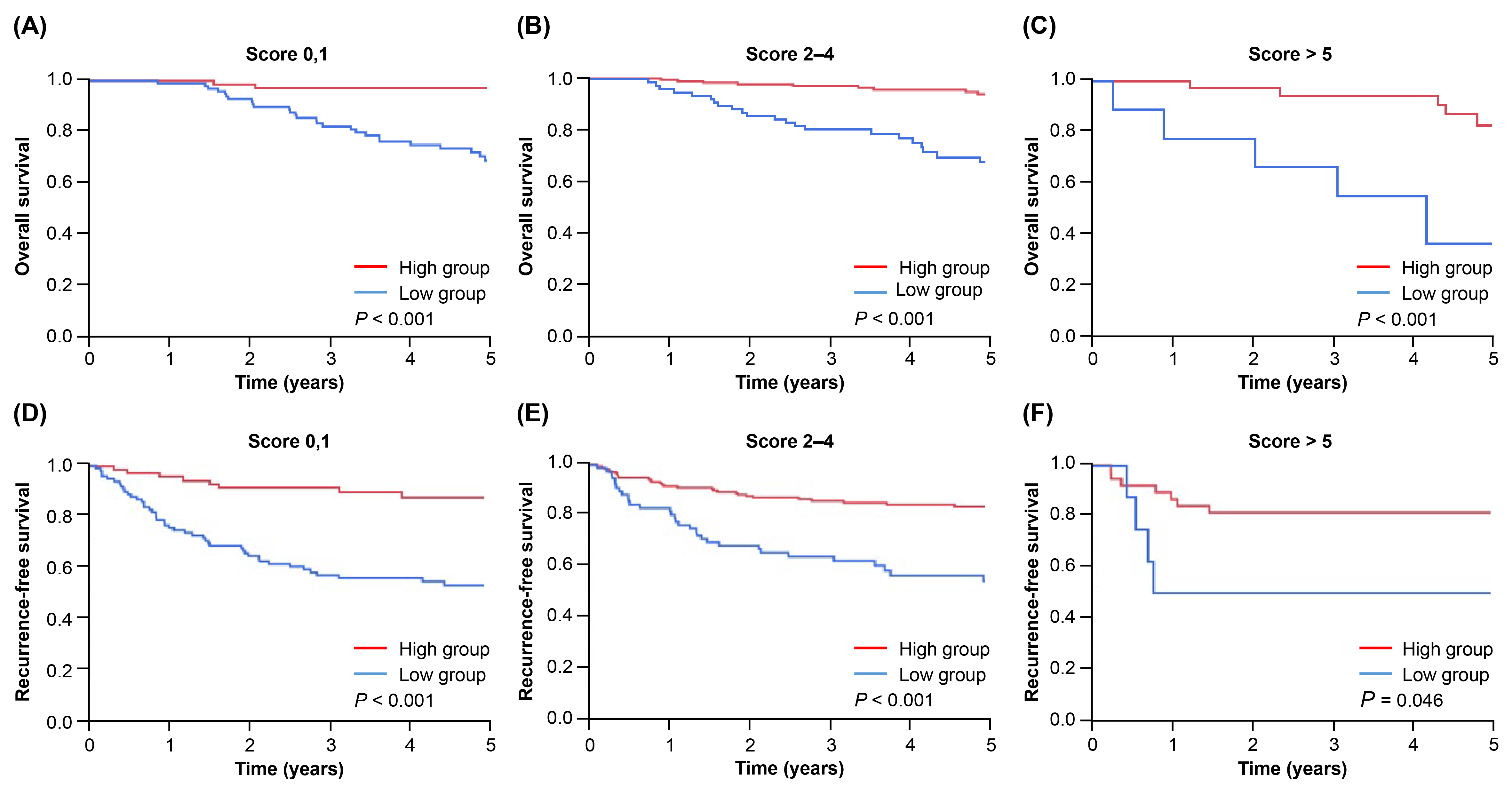

Supplement: Supplementary file 2 — Figure S2. [file AGS3-8-817-s001.tif]

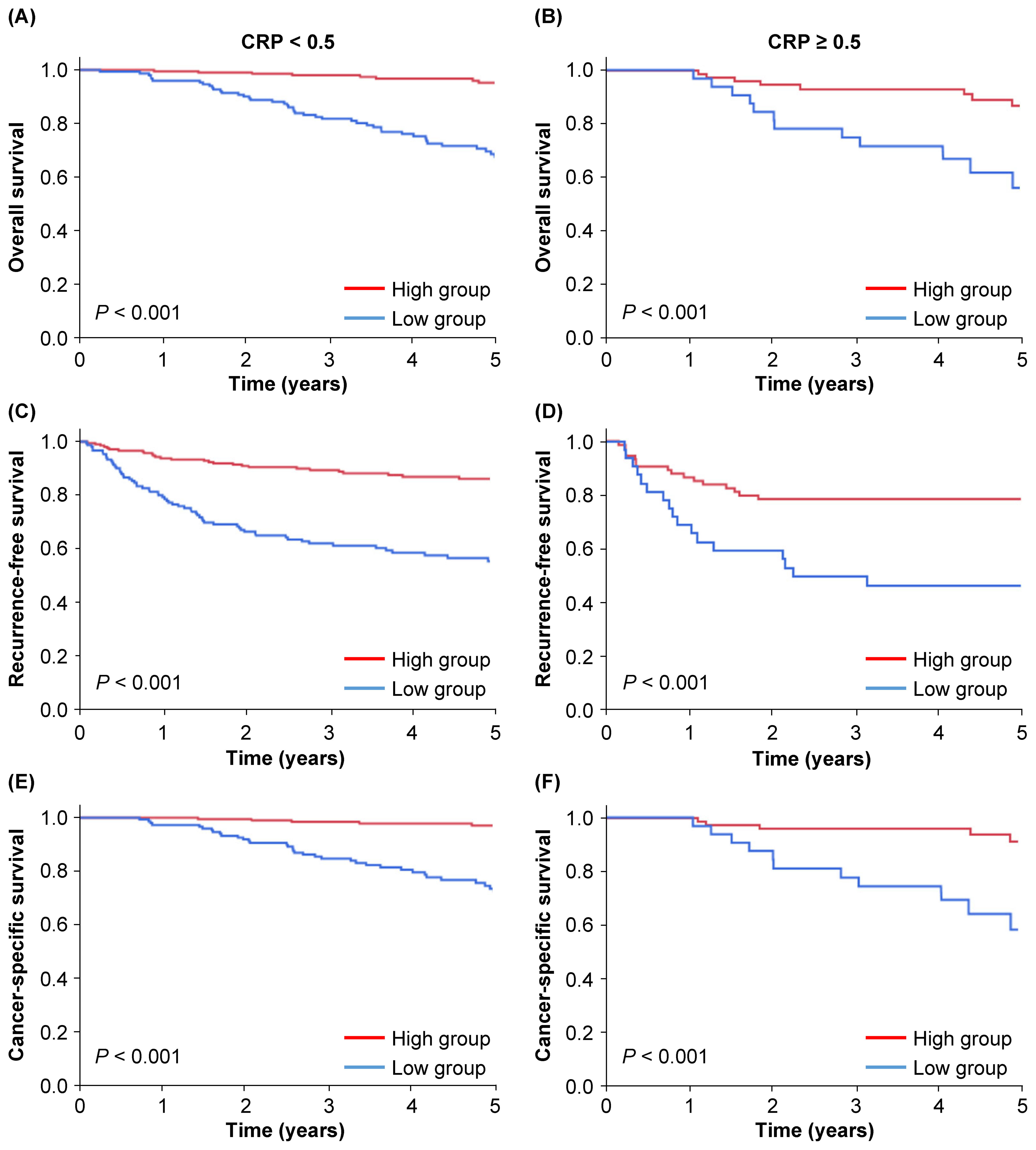

Supplement: Supplementary file 3 — Figure S3. [file AGS3-8-817-s003.tif]

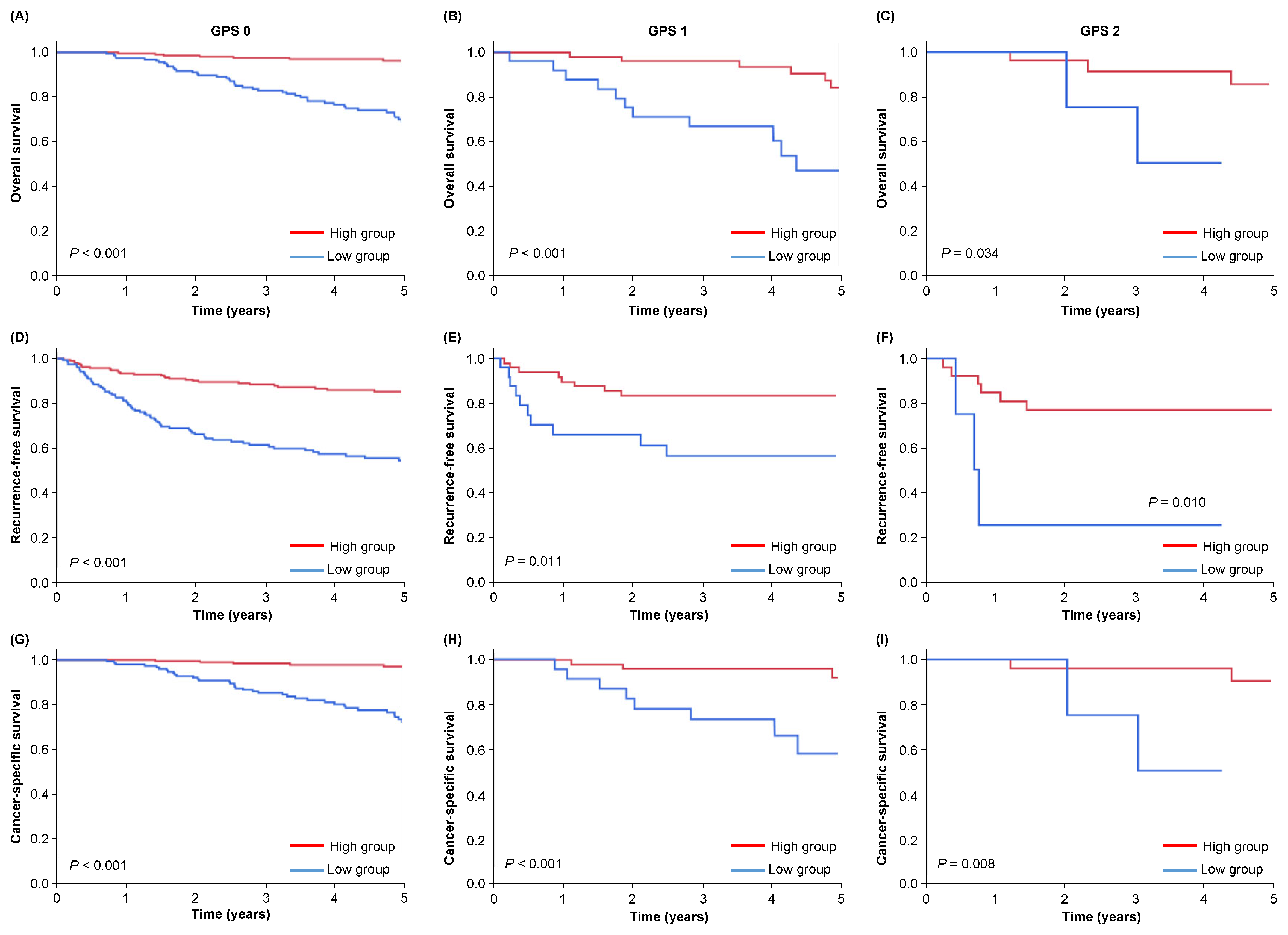

Supplement: Supplementary file 4 — Figure S4. [file AGS3-8-817-s004.tif]
