# Supplementary material for: Impact of perioperative prognostic nutritional index changes on the survival of patients with stage II/III colorectal cancer
Source: Ann Gastroenterol Surg. 2024 May 30;8(5):817–25. doi: 10.1002/ags3.12826 (PMC11368507; doi:10.1002/ags3.12826)
Supplement: Supplementary file 5 — Table S1. [file AGS3-8-817-s002.docx]

**Table S1_suppinfo.** Results of multivariate analyses of factors affecting recurrence

|  | **Multivariable analysis** | | |
| --- | --- | --- | --- |
|  | **HR** | **95% CI** | ***P*-value** |
| **Age, ≥70 years** | 0.813 | 0.546–1.209 | 0.307 |
| **Gender, male** | 1.220 | 0.847–1.759 | 0.286 |
| **Low group based on perioperative changes in PNI** | 3.833 | 2.623–5.560 | <0.001 |
| **LN dissection, D3** | 1.266 | 0.837–1.916 | 0.264 |
| **Histological type, poor/undifferentiated** | 1.643 | 0.905–2.981 | 0.102 |
| **Pathological T stage, T4** | 2.293 | 1.516–3.467 | <0.001 |
| **Pathological N Stage, N +** | 2.905 | 1.890–4.466 | <0.001 |
| **Adjuvant chemotherapy, yes** | 0.762 | 0.509–1.140 | 0.762 |

HR, hazard ratio; CI, confidence interval; PNI, prognostic nutritional index; LN, lymph node

**Table S2_suppinfo.** Multivariate analyses indicating factors affecting cancer-specific survival

|  | **Multivariable analysis** | | |
| --- | --- | --- | --- |
|  | **HR** | **95% CI** | ***P*-value** |
| **Age, ≥70 years** | 1.154 | 0.673–1.978 | 0.603 |
| **Gender, male** | 1.227 | 0.734–2.036 | 0.428 |
| **Low group based on changes in perioperative PNI** | 8.603 | 4.537–16.315 | <0.001 |
| **LN dissection, D3** | 1.134 | 0.639–2.010 | 0.667 |
| **Histological type, poor/undifferentiated** | 2.229 | 0.964–5.155 | 0.061 |
| **Pathological T stage, T4** | 1.966 | 1.114–3.471 | 0.020 |
| **Pathological N Stage, N +** | 5.259 | 2.583–10.708 | <0.001 |
| **Adjuvant chemotherapy, yes** | 0.801 | 0.462–1.389 | 0.430 |

HR, hazard ratio; CI, confidence interval; PNI, prognostic nutritional index; LN, lymph node
